# Resting-state fractal brain connectivity is associated with impaired cognitive performance in healthy aging

**DOI:** 10.1007/s11357-023-00836-z

**Published:** 2023-07-17

**Authors:** Akos Czoch, Zalan Kaposzta, Peter Mukli, Orestis Stylianou, Andras Eke, Frigyes Samuel Racz

**Affiliations:** 1 Department of Physiology, Semmelweis University, Budapest, Hungary; 2 Oklahoma Center for Geroscience and Healthy Brain Aging, University of Oklahoma Health Sciences Center, Oklahoma City, OK USA; 3 Vascular Cognitive Impairment and Neurodegeneration Program, Department of Neurosurgery, University of Oklahoma Health Sciences Center, Oklahoma City, OK USA; 4 International Training Program in Geroscience, Doctoral School of Basic and Translational Medicine/Department of Public Health, Semmelweis University, Budapest, Hungary; 5 Institute of Translational Medicine, Semmelweis University, Budapest, Hungary; 6 Berlin Institute of Health at Charité, University Hospital Berlin, Berlin, Germany; 7 Department of Neurology With Experimental Neurology, Charité-University Hospital Berlin, Corporate Member of Freie Universität Berlin and Humboldt Universität Zu Berlin, Berlin, Germany; 8 Department of Radiology and Biomedical Imaging, Yale University School of Medicine, New Haven, CT USA; 9 Present Address: Department of Neurology, Dell Medical School, The University of Texas at Austin, Austin, TX USA; 10 Mulva Clinic for the Neurosciences, Dell Medical School, The University of Texas at Austin, Austin, TX USA

**Keywords:** Fractal connectivity, Aging, Cognitive decline, Electroencephalography

## Abstract

Aging affects cognitive functions even in the absence of ongoing pathologies. The neurophysiological basis of age-related cognitive decline (CD), however, is not completely understood. Alterations in both functional brain connectivity and in the fractal scaling of neuronal dynamics have been linked to aging and cognitive performance. Recently, fractal connectivity (FrC) has been proposed — combining the two concepts — for capturing long-term interactions among brain regions. FrC was shown to be influenced by increased mental workload; however, no prior studies investigated how resting-state FrC relates to cognitive performance and plausible CD in healthy aging. We recruited 19 healthy elderly (HE) and 24 young control (YC) participants, who underwent resting-state electroencephalography (EEG) measurements and comprehensive cognitive evaluation using 7 tests of the Cambridge Neurophysiological Test Automated Battery. FrC networks were reconstructed from EEG data using the recently introduced multiple-resampling cross-spectral analysis (MRCSA). Elderly individuals could be characterized with increased response latency and reduced performance in 4–4 tasks, respectively, with both reaction time and accuracy being affected in two tasks. Auto- and cross-spectral exponents — characterizing regional fractal dynamics and FrC, respectively, — were found reduced in HE when compared to YC over most of the cortex. Additionally, fractal scaling of frontoparietal connections expressed an inverse relationship with task performance in visual memory and sustained attention domains in elderly, but not in young individuals. Our results confirm that the fractal nature of brain connectivity — as captured by MRCSA — is affected in healthy aging. Furthermore, FrC appears as a sensitive neurophysiological marker of age-related CD.

**Supplementary Information:**

The online version contains supplementary material available at 10.1007/s11357-023-00836-z.

## Introduction

Adverse impact of healthy aging leads to impaired functioning of the cardiovascular, skeletomuscular, respiratory, or immune systems [1–3]. Besides, of particular importance is the natural decline in cognitive capabilities, that occurs even in healthy aging [4, 5], i.e., when no other pathology is present [6, 7]. Aging-related cognitive decline not only affects a multitude of daily activities — and thus has a substantial effect on the quality of life —, but also bears psychological and socioeconomical implications [8]. Furthermore, even as of today it is a challenging task to differentiate naturally occurring loss of cognitive capabilities from early dementia or developing mild cognitive impairment [9]. This is a particularly important issue, as early detection of these conditions is key for plausible intervention strategies to take maximal effect. Accordingly, many studies set out to identify neurophysiological markers associated with various aspects of healthy aging; however, very few biomarkers have been identified yet that could be robustly linked with declining cognition [10].

It has been widely recognized that the brain’s capability of performing complex functions emerges from cooperation among its distributed neuronal assemblies. Accordingly, assessing the functional connectivity (FC) — i.e., how various brain regions interact with each other [11] — of the brain has received significant attention in the past decades, aiming for a better understanding of the system-level neurophysiological background of mental processes and functioning [12]. Previous studies have identified distinct sets of brain regions that form functional networks by their coordinated activity, such as the default mode network [13], the task positive network [14], or the dorsal and frontotemporal attention networks [15]. Such research is relevant in understanding aging-related alterations in cognitive functioning from multiple aspects.

Numerous studies have shown and confirmed that the topology of functional networks in the aging brain is different from those in a younger age [16–18]. The exact nature and extent of such alterations are yet to be fully understood; however, it is suggested that they reflect the combined effects of cell and thus function loss, along with emerging compensatory mechanisms [19, 20]. Moreover, some associations have also been found between these resting-state FC patterns and cognitive performance in elderly individuals [21–23]. FC was found altered in many clinical conditions [24] — developing predominantly in the elderly — that severely affect cognitive functioning, such as Alzheimer’s Disease, Parkinson’s Disease, or Mild Cognitive Impairment, and symptom severity was associated with connectivity metrics [25–27]. On a different note, a line of previous works — utilizing a vast array of imaging modalities — consistently reported that functional networks reorganize in response to increased mental workload or during solving various tasks [28–32]. This phenomenon might be understood as during task completion, irrelevant connections should be pruned to reduce wiring cost, while those that are required specifically for the given task should be activated [33]. It was also indicated that this task-related reorganization might be different in the elderly when compared to younger individuals, possibly reflecting the aging brain’s reduced ability to cope with increased workload [34]. All these considerations suggest that FC might be able to provide the much-needed insight into aging-related cognitive changes and their neurophysiological basis. However, as of today such markers are scarce, as only a handful of studies investigated how connectivity patterns that discriminate between young and elderly individuals might relate to the differences that can be observed in their cognitive capabilities [20, 35]. In this study, our goal was to take a step forward in this direction and identify such biomarkers, using the toolset of fractal connectivity (FrC), as explained below.

Fractal dynamics refers to the long-term (power-law decaying) autocorrelation observable in neural fluctuations [36, 37]. Many previous studies confirmed that neural activity exhibits fractal temporal scaling in its dynamics, which can be characterized via a scaling exponent either in the frequency or in the time domain [38]. Even though the precise neural basis of this scale-free nature is not fully understood, it has been shown that the fractal scaling exponent is altered under multiple conditions, such as sustained attention [39], psychiatric conditions such as schizophrenia [40], or aging [41–43]. Recently, however, it has been shown that this phenomenon is not only observable in regional neural activity, but in the FC among various brain regions as well [44]. In fact, fractal scaling of FC dynamics has been identified via multiple imaging modalities and approaches [45–49]. Furthermore, it has been observed lately that FrC patterns also change in response to increased mental workload evoked by a pattern recognition test [50], indicating a relationship between FrC and cognitive functioning. In general, one can take two approaches for FrC assessment: (*i*) estimate connectivity in a time-resolved manner and then identify fractal scaling in the fluctuations of the obtained measures [51–54] or (*ii*) compute scale-free coupling directly and characterize the fractal nature with the bivariate scaling exponent [55–58]. Numerous methods have been developed for both approaches; however, these mostly expect purely fractal signals as inputs. On the other hand, neural activity rather appears to be a mixture of scale-free “background” activity and oscillatory components with characteristic frequencies, such as theta or alpha oscillations in electroencephalography (EEG) recordings [59]. The presence of such components can bias the estimation of the auto- and the cross-spectral slope. To overcome this issue, Wen and Liu [60] introduced a technique — based on the works of Yamamoto and Hughson [61, 62] — termed irregular resampling auto-spectral analysis (IRASA) to separate the fractal and oscillatory components in the auto-power spectrum, thus allowing for unbiased estimation of the univariate fractal scaling exponent. Since then, IRASA has been extended to the bivariate domain; we recently introduced multiple-resampling cross-spectral analysis (MRCSA), which allows for unbiased fractal characterization of not only regional neural activity, but also fractal connectivity networks [63].

Summarizing previous evidence, FC and fractal dynamics of neural activity both appear to be affected in aging [18, 41, 43]. Furthermore, these neurophysiological characteristics also showed associations with cognitive functions [39, 50, 59]; therefore, we hypothesized that an approach that combines these two concepts — in fractal connectivity analysis — might provide sensitive neural markers that can link age-related changes in brain network organization to reduction in cognitive capabilities. To verify this, we analyzed resting-state fractal connectivity networks — as reconstructed from EEG data using MRCSA — of healthy elderly individuals and compared them with those of a young control group. Furthermore, all participants completed a comprehensive cognitive battery including tasks that are sensitive for age-related cognitive impairment and dementia. Our results not only indicate a decline in cognitive performance and alterations of FrC in the elderly group, but we also found that many of these discriminative connections expressed strong relationships with performance measures in various cognitive domains.

## Materials and methods

### Participants and measurement protocol

In total, 47 healthy volunteers were recruited in this study; 25 young (aged between 18 and 35 years, mean = 25.7, 12 females) and 22 elderly (over the age of 60, mean = 66.2, 8 females). The study was conducted according to the standards of the Declaration of Helsinki and was approved by the Semmelweis University Regional and Institutional Committee of Science and Research Ethics (approval number: 2020/6). All subjects provided written informed consent prior to the measurement. Participants were instructed not to take any substances that could affect cognitive performance (e.g., caffeine) for at least 3 h prior to measurement, and to have at least 6 h of sleep the previous night. Exclusion criteria included neuropsychological or psychiatric morbidities, history of brain damage, current medication affecting the central nervous system, pregnancy, or the presence of any general medical condition (e.g., severe cardiovascular pathologies). Although all subjects were able to complete the measurement protocol, one young and three elderly subjects were later excluded from further analysis due to excessive head motion and/or poor signal quality (see below), resulting in a final sample size of 24 young (age: 25.37 ± 3.20) and 19 elderly (age: 66.39 ± 6.09) subjects, a total of 43.

Recordings took place in a quiet, darkened room in the Department of Physiology at Semmelweis University. Participants were seated in a comfortable chair in front of a 24-inch computer display during the measurement (approximately 0.8 m from the screen) and were asked to refrain from movements and facial expressions, in order to minimize the number of signal artifacts. Measurement protocol and subsequent analyses were designed and implemented in MATLAB (Mathworks, Natick, MA, USA). The session began with a 3-min-long, eyes-closed (EC) resting-state period, followed by an eyes-open resting-state period of same length. Note that the full recording session then proceeded to continue with 3 different cognitive paradigms (visual pattern recognition, n-back, and maze paradigm) for approximately 1 h; however, in the current study, we limited our analyses to EEG data collected in EC resting state.

### CANTAB

Baseline assessment of cognitive performance was carried out after completing the EEG recording session, using seven cognitive tests of the *Cambridge neuropsychological test automated battery* (CANTAB)*.* Originally developed by the University of Cambridge, CANTAB serves as validated and precise measures of various aspects of cognition. CANTAB tests include tasks of working, visual and spatial memory, learning and executive function, reaction time, information processing, and many others, also providing the information in which condition the given test might be most indicative. From the available palette, we selected seven tasks that are associated with age-related cognitive decline and dementia [64], namely: motor screening task (MOT), delayed match to sample (DMS), paired associates learning (PAL), immediate and delayed pattern recognition memory (PRM), reaction time (RTI), rapid visual processing (RVP) and spatial working memory (SWM). A short description of each task is provided in Table 1, while for more detailed information and illustration the reader is referred to the official website (https://www.cambridgecognition.com/cantab/).

**Table 1:** Applied cognitive tests

Name	Description (time in minutes)	Assessed functions	Outcome
MOT	Colored crosses appear on the screen in different locations and the participant must tap on them as fast as possible. (**2**)	Sensorimotor skills	Reaction time and precision
DMS	A complex visual pattern is presented to the participant. After a brief delay (0, 4, or 12 s), four patterns are shown, the original and three similar. The participant must choose the original pattern from the four. (**7**)	Visual matching ability, short-term visual recognition memory	Response latency, number of correct selections
PAL	Boxes are displayed on the screen and the participant opens them in arbitrary order. One or more contains a visual pattern. After opening every box, the patterns are displayed in the middle of the screen one by one, and the participant must choose the box the pattern originated from. (**8**)	Visual memory and learning	Number of errors, number of attempts and first attempt memory score
PRM	A series of visual patterns — designed to be hardly verbally describable — are shown to the participant. In the first phase the participant is presented with two patterns, one shown before and a novel one. They must choose the pattern which was presented previously. A second phase of the same task is administered after a delay of ~ 20 min (**4** and **4**)	Visual pattern recognition memory	Response latency, number, and percentage of correct responses
RTI	In the starting position the participant holds down a button at the bottom of the screen. One or five circles are presented above, and a yellow dot appears in one of them after a random waiting time. The participant must react as fast as possible by releasing the home button and touching the yellow dot. (**3**)	Motor and mental response speed, response accuracy and impulsivity	Reaction time, movement time and number of errors
RVP	Digits from 2–9 are presented in a pseudorandom order (100 digits/min). The participant must detect a pre-defined target sequence (e.g., 2–7-3) and respond by tapping a button as fast as possible. Difficulty can change when participants must watch for multiple target sequences at the same time. (**7**)	Sustained attention	Response latency, correct responses, probability of false alarms and sensitivity
SWM	Several boxes are shown on the screen. By selecting the boxes, the participant must search for a yellow token. The boxes are closed again after selecting them. Depending on the difficulty level, the number of boxes can be increased to 12. (**4**)	Retention and manipulation of visuospatial information, working memory and problem solving strategy	Between errors, within errors, total errors and strategy

*MOT* motor screening task, *DMS* delayed matching to sample, *PAL* paired associates learning, *PRM* pattern recognition memory, *RTI* reaction time, *RVP* rapid visual information processing, *SWM* spatial working memory

Participants completed the CANTAB session using a 10.2″ iPad tablet computer (to which the tasks were standardized) using their dominant hand. All tasks in the battery started with an instructional introductory session that could be set to the user’s native language (Hungarian or English in this study), and the participant could only proceed to complete the test after finishing the training, ensuring a firm understanding of the upcoming task. In order to minimize the possibility of any help or bias from the investigators, participants completed the CANTAB assessment alone in a separate room, which took approximately 40 to 50 min.

### Data acquisition and pre-processing

EEG data were recorded using an Emotive Epoc+ wireless device with the corresponding EmotivPRO software (Emotiv Systems Inc., San Francisco, CA, USA). Activity of 14 brain regions (10–10 standard montage locations: AF3, AF4, F3, F4, F7, F8, FC5, FC6, T7, T8, P7, P8, O1, and O2, CMS, and DRL reference electrodes at P3 and P4) were monitored with a sampling rate of 256 Hz. Data collection only began after confirming maximal contact quality, as indicated by the EmotivPRO software. The device had an internal sampling rate of 2048 Hz and an effective bandwidth between 0.2 and 45 Hz (achieved by a 5th order Sinc filter and notch filters at 50 and 60 Hz). Raw data was down-sampled to 256 Hz and then forwarded wirelessly to a desktop computer.

EEG data was first visually inspected after applying an additional 4th order, zero-phase Butterworth filter with cutoff frequencies 0.5 and 45 Hz. Then, continuous artifact-free epochs were selected for further processing by two investigators. Only segments that were assessed by both researchers independently as absent of extensive artifacts were included, resulting in a final epoch length of 72 s (the shortest length available from all participants). As described previously, four subjects (1 from the young and 3 from the elderly group) were excluded at this stage from further analysis, where such a segment could not be identified. Independent component analysis (ICA) was performed using the EEGLAB toolbox [65] on data trimmed to equal length. Independent components were inspected manually, and those associated with eye movements, skeletal muscle activity or other sources of noise were identified and removed before performing reverse ICA. Finally, data was re-referenced to the common average electrode.

### Estimating fractal connectivity – MRCSA

Multiple-resampling cross-spectral analysis (MRCSA) is a recently developed method designed to separate the fractal component in the cross-spectrum of two signals from those related to oscillatory components [63]. The technique is the bivariate extension of the previously introduced irregular resampling auto-spectral analysis (IRASA) by Wen and Liu [60], which allows for separating the fractal component in the auto-power spectrum of a single time series. Here, we provide a brief introduction to the main concepts behind both methods, while for detailed descriptions the reader is referred to the original publications.

IRASA and MRCSA both exploit the self-affine property of fractal signals [66]. Namely, given a fractal time series $f\left(t\right)$ and its rescaled (resampled) version ${f}_{h}\left(t\right)$ with scaling factor $h$, the self-affine property can be formulated as

1 $${f}_{h}\left(t\right)\triangleq {h}^{H}f\left(t\right),$$

where $H$ is the Hurst-exponent characterizing the fractal scaling property. This relationship also holds in the frequency domain, namely that the amplitude of the rescaled signal at angular frequency $\omega$ (${F}_{h}\left(\omega \right)$) corresponds to that of the original ($F\left(\omega \right)$) rescaled by factor ${h}^{H}$ [60]:

2 $${F}_{h}\left(\omega \right)\triangleq {h}^{H}F\left(\omega \right).$$

Note that fractal signals have a broadband power distribution, where the squared amplitude is inversely proportional to the frequency, and the relationship is established via the scaling exponent $\beta$ as

3 $${\left|F\left(\omega \right)\right|}^{2}\propto c\cdot {\omega }^{-\beta }$$

with constant $c$. Also, since $H$ and $\beta$ capture the same fractal property, they are also inherently related [36, 37]. Importantly, if $f\left(t\right)$ is up- and down-sampled by factors $h$ and $1/h$ (yielding ${f}_{h}\left(t\right)$ and ${f}_{1/h}\left(t\right)$, respectively), the geometric mean of their power spectra indeed returns that of the original signal:

4 $$\sqrt{{h}^{H}F\left(\omega \right)\cdot \frac{1}{{h}^{H}}F\left(\omega \right)}=F\left(\omega \right)$$

In contrast, oscillatory signals are characterized by spectra, where the power is only non-zero at the characteristic frequency and its harmonics [61, 62], while zero elsewhere. Consequently, by resampling an oscillatory signal, the peaks corresponding to harmonic oscillations only get “relocated” according to $h$ (and equivalently for $1/h$), and taking the geometric mean only attenuates these without allowing for recovery of the original spectrum [61, 62]. This provides the means to separate the fractal element of the spectrum in case of a composite signal that is comprised of fractal and oscillatory components, assuming their independence. Precisely, by performing the above-described procedure for a set of non-integer rescaling factor pairs and taking the median over the obtained geometric mean spectra, one can recover the fractal component of the power spectrum without the biasing contribution of oscillatory peaks [60]. Finally, by subtracting the fractal component from the original (mixed) power spectrum, one can also obtain an estimate of the spectrum of the oscillatory signal component.

It has been shown that the same principles can be applied to obtain the fractal component in the cross-spectrum of two coupled time series [63]. In that, given two time series $x\left(t\right)$ and $y\left(t\right)$ the fractal component of the cross-spectrum can be separated utilizing MRCSA, then the cross-spectral slope, ${\beta }_{XY}$, can be estimated without the biasing effect of oscillatory components [63]. The cross-spectral slope gives an equivalent description of the fractal scaling property as the bivariate Hurst exponent [57] and indicates how the long-term coupling of two processes behaves in relation to time scale. Therefore, in this study we utilized MRCSA and the obtained ${\beta }_{XY}$ exponents to characterize fractal connectivity between disparate brain regions, while integrated cross-spectral slope of the fractal, oscillatory, and mixed spectra were used to describe functional connectivity of fractal, oscillatory, and overall brain activity, respectively.

As neural activity recorded by EEG is widely considered non-stationary on the long scale (Boutros et al., 2008), the 72-s-long, cleansed EEG segments were first divided into non-overlapping epochs of 8 s (yielding 9 epochs), and fractal connectivity analysis was performed on these shorter data sets separately. MRCSA was carried out with the following parameters: spectral power was computed between effective frequencies 2 and 22.5 Hz (see below) with a resolution of 0.128 Hz, and rescaling factors $h$ (and their corresponding reciprocals $1/h$) were utilized in the range of 1.1 to 1.9 in 0.05 increments, as according to Wen and Liu [60]. The analysis and subsequent spectral slope estimation was performed in the frequency range 2 to 22.5 Hz, as the resampling procedure in IRASA (and MRCSA) also carries over the effect of any previously applied filtering procedures proportionally to the utilized rescaling factors, thus diminishing the effective frequency range for analysis [40]. Estimates of ${\beta }_{XY}$ and ${\beta }_{X}$ were obtained using ordinary least squares regression of log cross-spectral power on log frequency. This yielded 14-by-14 matrices for ${\beta }_{XY}$ for each epoch. Note that even though spectral exponents were found negative given the inverse relationship between auto- and cross-spectral power and frequency, in the following we report slopes as positive (i.e., after multiplying by − 1) according to convention [37]. Integrated spectral power was computed in the broadband frequency range ($2-22.5$ Hz) by summing power between the boundary frequencies, yielding a 14-by-14 matrix for each spectrum type (fractal, oscillatory, and mixed) in each epoch. Finally, corresponding matrices were averaged over the 9 epochs to obtain robust estimates of resting-state functional connectivity in each measure.

### Statistical analysis

CANTAB scores were compared between the young and elderly groups using two sample, unpaired tests. Precisely, normal distribution of the data was first verified by Lilliefors test, then a two-sample t test was applied in case of normality, while a Mann–Whitney *U* test otherwise. Due to many CANTAB output measures capture essentially the same information (e.g., mean, median or mode of the number of attempts) thus unnecessarily increasing the number of comparisons (${n}_{CANTAB}=154$), results were adjusted using the less conservative false discovery rate (FDR) method of Benjamini and Hochberg [67].

Estimates of connectivity were compared connection-by-connection using two-sample tests between the young and elderly groups, following the same pipeline, as described with CANTAB scores previously. In order to prune for more characteristic differences, obtained results were adjusted for multiple comparisons using Bonferroni’s method for each connectivity metric separately (${n}_{conn}=\left(14\cdot 13\right)/2=91$).

Finally, we performed an exploratory analysis to see if those CANTAB measures that indicated difference in cognitive capabilities between the young and elderly groups showed any relationship between those connections that were identified as implying different fractal connectivity. For this purpose, we computed the Spearman cross-correlation coefficient between connectivity measures and CANTAB scores in the two groups, separately. Due to the sheer amount of comparisons (${n}_{comb}=2\cdot 17\cdot 54$) and relatively small sample size, we report these outcomes without adjusting for multiple comparisons (that would render most of the results statistically non-significant), and thus one must employ caution when interpreting these results.

## Results

### Behavioral results

No significant differences were found in the MOT task, confirming that all participants had the sufficient sensorimotor skills to complete the remaining tasks, as well as determining that sensorimotor skills were unlikely to confound any plausible contrast between the two groups. For the remaining cognitive tests, we found significant differences between the CANTAB scores of young and elderly groups in 54 cases following FDR adjustment. Precisely, in 10 measures for DMS, 16 for PAL, 4–4 for PRM, and RTI, 8 for RVP, and 12 for SWM tasks. We summarize the results below (all *p*-values FDR-adjusted), while a detailed report is provided in Supplementary Table S1.

In the DMS task — where subjects were presented a visual pattern, then they had to identify the previously shown pattern among a set of now ones presented after the original image with various latencies — we found the response time increased in the elderly when compared to the young group when the target and response stimuli were shown simultaneously (median correct latency, simultaneous, young: 2.25 s, elderly: 3.24 s, *p* = 0.0010) or with various delays (mean correct latency, all delays, young: 2.49 s, elderly: 3.96 s, *p* = 0.0184). We also found the standard deviation of response times in the 4-s delay condition increased in the elderly group (*p* = 0.0427). However, no significant differences were found in performance, i.e., in the proportion of correct/erroneous responses.

We found the most significant differences (namely, 16) regarding the PAL task, which challenged the visuo-spatial memory of the subject, as they had to recall pattern locations. These results demonstrated a general drop in performance in the elderly group across most task conditions (recollection of 4, 6, 8, and 12 patterns). General first attempt memory score (PALFAM28, see Supplementary Table S1 for definition) was found to be 11.9474 in the elderly, compared to the 16.5417 in the young group (*p* = 0.0004). More detailed, the number of errors (and relatedly, the number of attempts) were higher in the elderly group for 4 (errors: *p* = 0.0251, attempts: *p* = 0.0241), 6 (errors: *p* = 0.0012, attempts: *p* = 0.0013), and 8 (errors: *p* = 0.0314) patterns. Overall, the elderly group could be characterized with decreased performance that was most pronounced in the easier tasks (4 and 6 patterns), but less for more difficult versions (8 and 12 patterns).

In the PRM task — testing pattern recognition memory —– we found an increased response latency both in the immediate (median correct latency, young: 1.28 s, elderly: 1.74 s, *p* = 0.0015) and in the delayed (median correct latency, young: 1.53 s, elderly: 1.93 s, *p* = 0.0033) conditions. No difference was found, however, in the percentage of correct responses between the two groups.

The RTI task challenged the response time and accuracy of subjects in case of one or five possible targets. Elderly participants made significantly more inaccurate responses (*p* = 0.0244), while we also found the mean (young: 0.34 s, elderly: 0.39 s) and standard deviation (young: 0.0365 s, elderly: 0.0469 s) of response times increased in the same group (*p* = 0.0022 and *p* = 0.0228 for mean and standard deviation, respectively).

The RVP task challenged the sustained attention and working memory of subjects. We found that elderly participants not only responded with a greater latency (median latency, young: 0.41 s, elderly: 0.53 s, *p* = 0.0002), but their overall performance (computed from hits/misses, regardless of latency, referred to as RVPA from here on) was also reduced (young: 0.9398, elderly: 0.8971, *p* = 0.0143).

The SWM was the most complex among the applied tests that not only challenged the participants’ spatial working memory but also assessed their strategy in solving the task. During a trial, subjects could make three types of mistakes: (i) between errors, where they revisit a box in which a token was already found, (ii) within errors, where they revisit a box that was already found empty, and (iii) total errors, where they revisit a box that is certain not to contain a token. In general, we found reduced performance (as indicated by increased between and total errors) in the elderly group for all difficulty levels (4, 6, 8, and 12 boxes, for details, see Supplementary Table S1). Furthermore, it was indicated that elderly participants applied less effective strategies when searching for the token when compared to young subjects (*p* = 0.0241).

In summary, the elderly group could be characterized with increased response latency in four of the investigated cognitive tests (DMS, PRM, RTI, and RVP), while their performance was reduced when compared to young individuals also in four tasks (PAL, RTI, RVP, and SWM). In two cases, increased response latency was accompanied by comparable task performance (DMS and PRM), while in two of the tasks the elderly group underperformed in both response time and accuracy (RTI and RVP). No difference was identified between the two groups in the MOT task.

### Differences in fractal connectivity

**Fig. 1:**
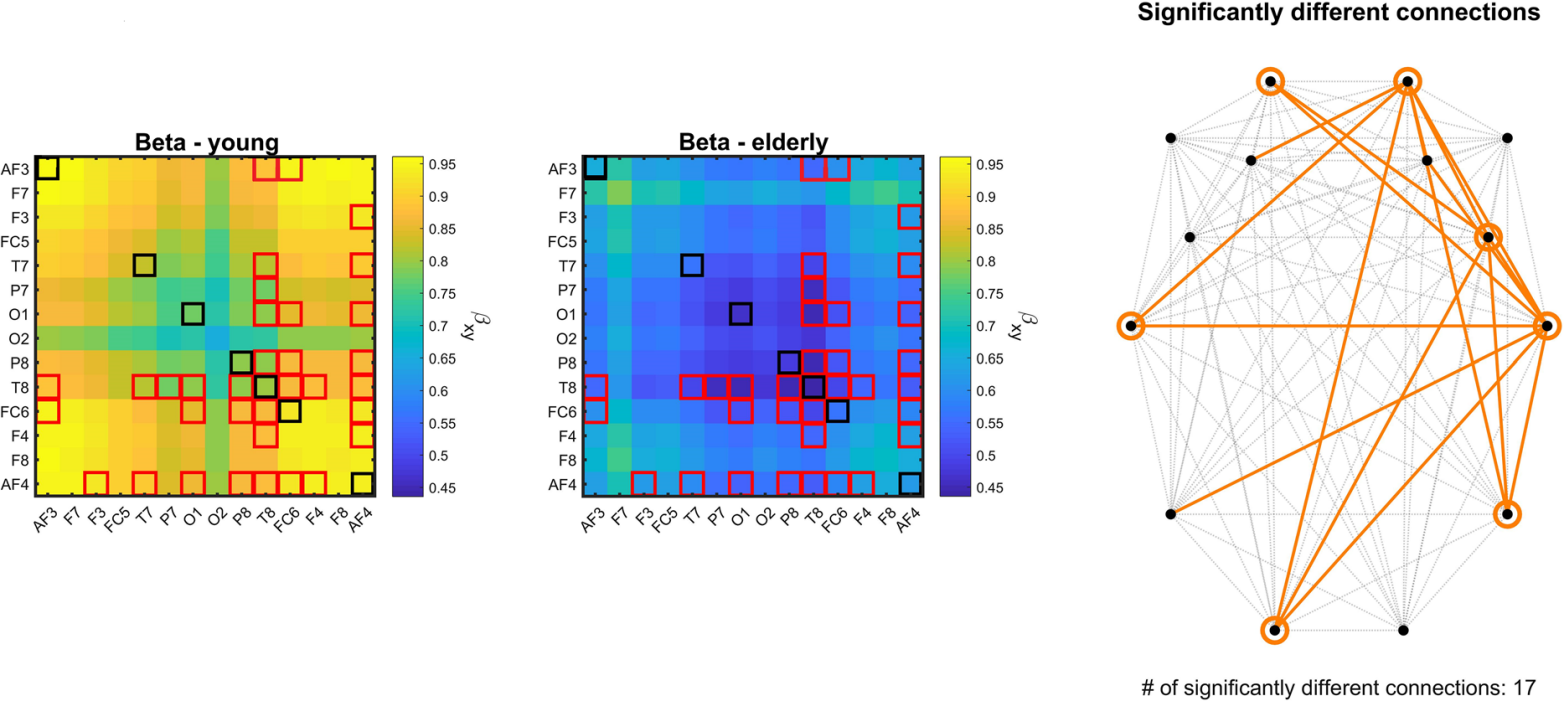
Auto- and cross-spectral exponents in the young and elderly groups. In the left and middle panels, black and red squares denote the locations/connections, where the spectral exponent was found significantly different between the two study groups. The right panel illustrates the cortical topology of these differences, where orange circles and lines denote the region/connection where the differences were found in the auto- and cross-spectral exponents, respectively

Cross-spectral slope of 17 connections was found reduced in the elderly group (Fig. 1). Additionally, the univariate spectral slope of seven cortical locations was also lower when compared to the young group. Although auto- and cross-spectral slopes were generally higher in younger individuals (Fig. 1, left compared to middle panel) over the entire cortex, significant connections were mostly linked to the right frontal and temporal areas. Correspondingly, regional auto-spectral slopes were found to be different mostly over the bilateral frontal and temporal regions, with also in the left occipital cortex (Fig. 1, right panel). Notably, all obtained cross- and auto-spectral exponents fall into the range of 0.45 and 0.95, indicating fractional Gaussian noise-type neural activity [36], as compared to white noise (slope = 0) or fractional Brownian motion (slope > 1).

We found no differences in either auto- or cross-spectral power in mixed, fractal, and oscillatory spectra between the two groups.

### Correlations between cognitive function and fractal connectivity

Finally, we explored if there are plausible relationships between fractal connectivity and cognitive performance. We reduced our analysis to those CANTAB scores and brain connections/regions that were identified to be different between young and elderly individuals (i.e., to the subset of variables discriminative between young and elderly), as our goal was to reveal correlations that might relate cognitive decline to neurophysiological processes in aging. We completed this analysis for the young and elderly groups separately.

Surprisingly, we only found sporadic relationships between cognitive performance and fractal connectivity (in the selected subset of variables) in the young group. Precisely, PAL mean error score was correlated with ${\beta }_{XY}$ of one connection (O1-FC6, *r* = 0.4363, *p* = 0.0330), while standard deviation of DMS latency was correlated with ${\beta }_{XY}$ of three connections (O1-AF4, *r* = 0.4070, *p* = 0.0495; FC6-AF4, *r* = 0.4470, *p* = 0.0297; F4-AF4, *r* = 0.4200, *p* = 0.0421). Additionally, spectral slope of AF4 was correlated with two measures (latency and standard deviation of DMS latency, *r* = 0.4409, *p* = 0.0322, and *r* = 0.4861, *p* = 0.0171, respectively) while that of T7 with the standard deviation of five-choice RTI latency (*r* =  − 0.4217, *p* = 0.0412).

On the other hand, in the elderly population, we found significant correlations between cross-spectral slope and CANTAB output measures in 59 cases, while an additional 26 cases for auto-spectral slope. After considering redundant measures (see below), most of these relationships with fractal connectivity were identified for RVP performance (RVPA) and PAL performance in the case of 6 patterns.

**Fig. 2:**
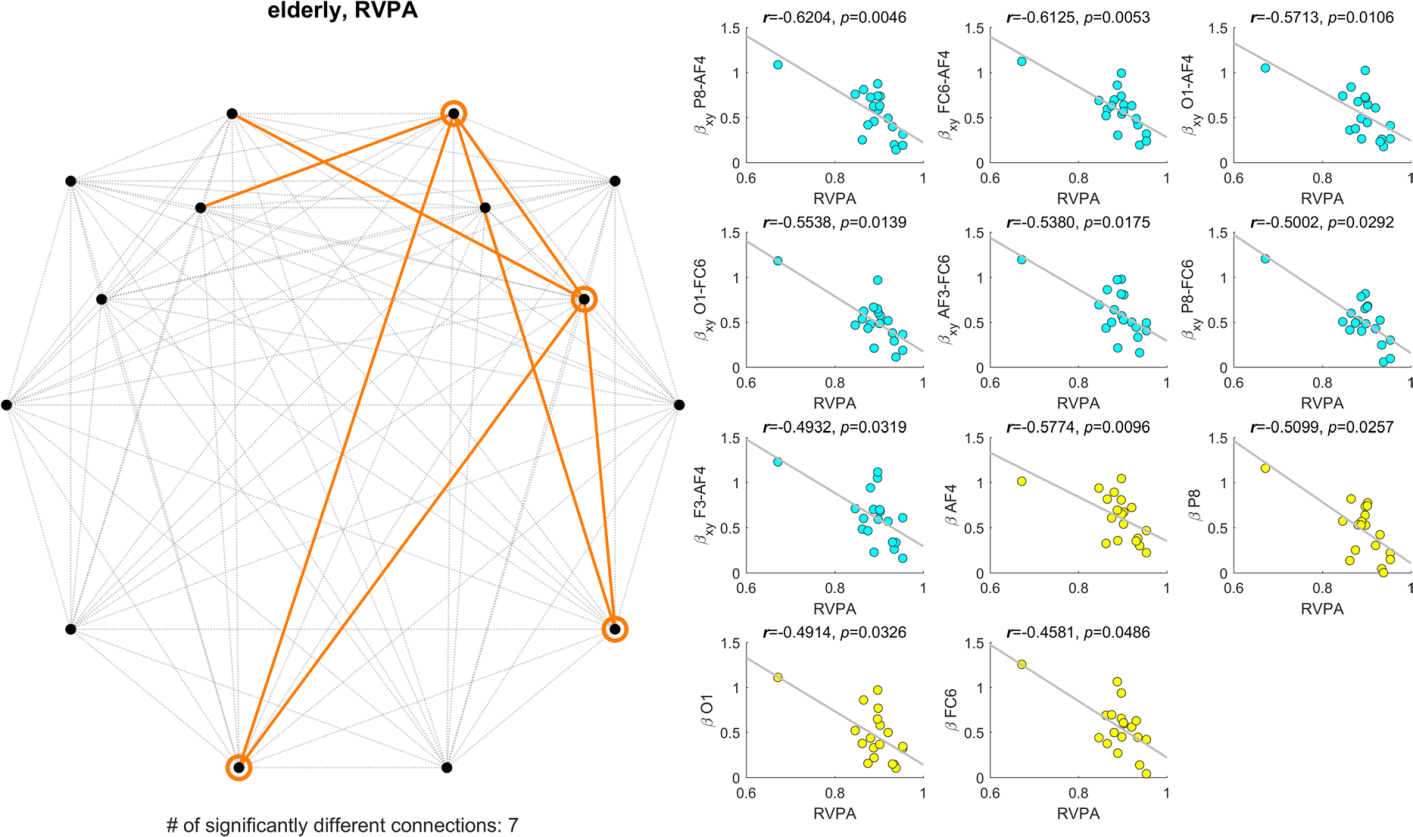
Significant correlations between spectral exponents and rapid visual processing scores (RVPA). The left panel indicates the locations (circles) and connections (orange lines) where ${\beta }_{X}$ or ${\beta }_{X,Y}$ expressed significant correlation with RVPA scores. Subplots of the right panel show the individual scatterplots for all relationships (yellow: ${\beta }_{X}$ vs. RVPA, blue: ${\beta }_{X,Y}$ vs. RVPA)

Figure 2 (left) shows the topology of connections where the cross-spectral slope correlated with the RVPA measure (7 cases), while rings indicate the cortical locations where the auto-spectral slope expressed a significant correlation (4 regions). Panels on the right of Fig. 2 detail the individual scatterplots illustrating the found relationships between the corresponding measures (blue for cross- and yellow for auto-spectral slope). Apparently, a lower spectral slope could be associated with a better performance in the RVP task, as indicated by the inverse relationship on the scatterplots. An important note must be made, however, regarding the scatter plots on the right panel; the found correlations might have been biased by one outlier sample. In order to explore this effect, we recomputed all correlations in this case after removing data from the given subject. We obtained comparable results, in that ${\beta }_{XY}$ of the first four connections listed (from P8-AF4 to O1-FC6) and ${\beta }_{X}$ of AF4 were found significantly correlated with RVPA (*p* < 0.05), while the rest of the relationships were rendered marginally significant (*p* < 0.10).

**Fig. 3:**
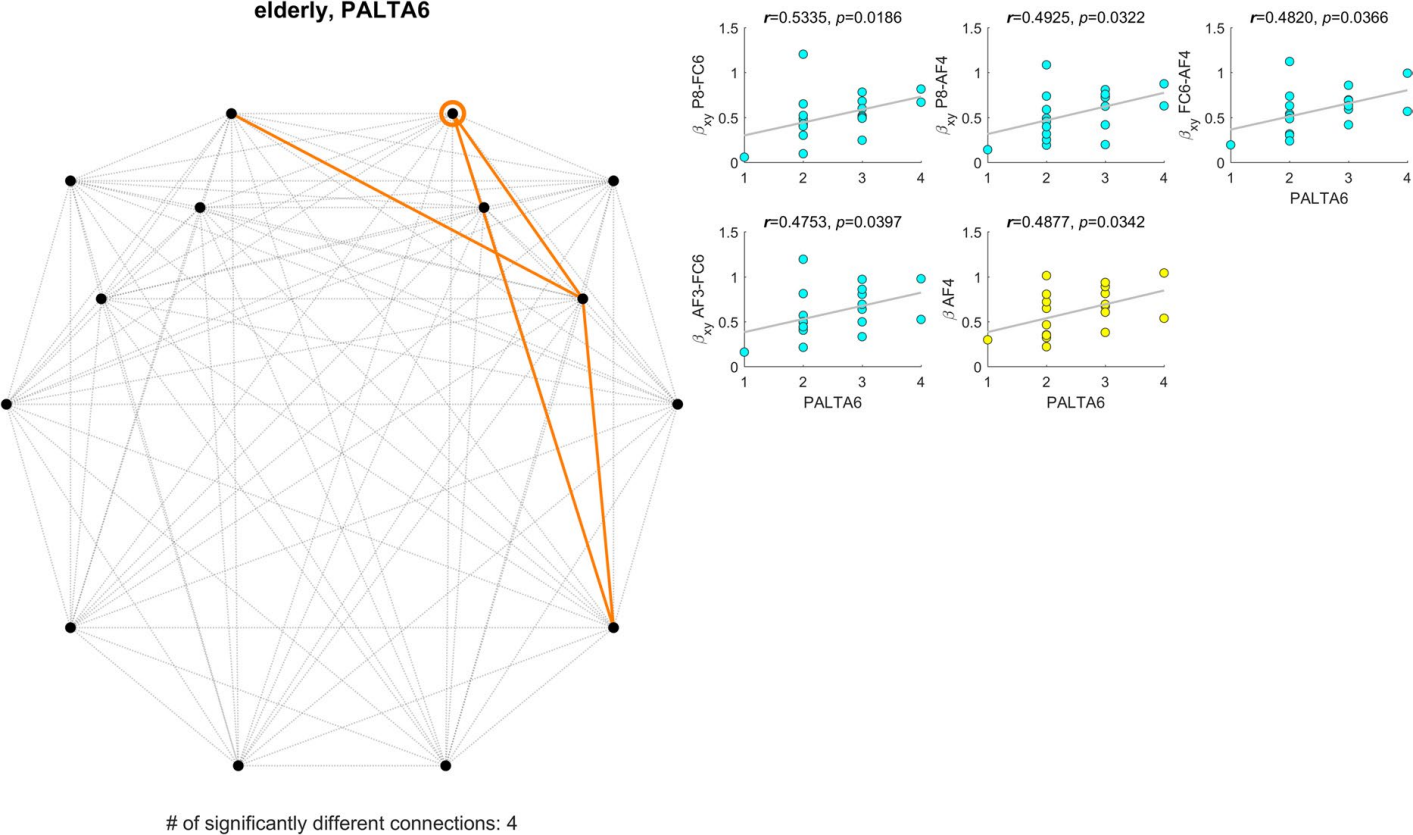
Significant correlations between spectral exponents and paired associates learning total errors in case of 6 patterns (PALTA6). The left panel indicates the locations (circles) and connections (orange lines) where ${\beta }_{X}$ or ${\beta }_{X,Y}$ expressed significant correlation with PALTA6 scores. Subplots of the right panel show the individual scatterplots for all relationships (yellow: ${\beta }_{X}$ vs. PALTA6, blue: ${\beta }_{X,Y}$ vs. PALTA6)

Similarly, Fig. 3 illustrates the relationships with the total number of attempts in the PAL task with 6 patterns in a similar fashion. In accordance with the previous results regarding RVPA, a higher spectral slope (4 connections and 1 cortical region) was associated with more attempts, i.e., worse performance.

As expected, we found almost identical results regarding the probability of hits in the RVP task (RVPPH, see Supplementary Fig. S1), as well as percentile-transformed and *z*-scored RVPA values (data not shown). Notably, RVPPH values show a more even distribution as compared to RVPA (regarding the outlier sample). Without this distorting effect we identified a strong relationship between performance in the RVP task and fractal connectivity/regional activity, as RVPPH was found significantly correlated to ${\beta }_{XY}$ of 6 connections and ${\beta }_{X}$ of 3 cortical regions (Supplementary Fig. S1), almost identical in topology to those presented in Fig. 2. Additionally, we found sporadic correlations between fractal connectivity and CANTAB scores in other tasks; however, these outcomes did not provide a coherent pattern. Details of these are presented in the Supplementary Material (Supplementary Figs. S2 and S3).

## Discussion

In this study, we assessed resting-state fractal connectivity in young and healthy elderly populations and investigated how such characteristics of neural activity relate to performance in a range of cognitive domains. Our analyses not only revealed altered resting-state FrC patterns in elderly individuals when compared to the cohort of young control participants, but many of the discriminative neural patterns showed strong association with cognitive functions that were found diminished in the elderly population. Therefore, these outcomes might provide a missing link between declining cognitive capabilities commonly observed in healthy aging and the plausible neural mechanisms behind them.

### Increased response latency and impaired cognitive performance in healthy aging

Instead of focusing on how connectivity patterns change in response to increased mental workload and how this response might be altered in aging, our aim was to identify patterns in resting-state activity that could be associated with (or even predict) cognitive performance. For this purpose, we intended to complete a broad evaluation covering multiple cognitive domains, concentrating especially on those mostly affected by aging [68, 69]. The selected set of seven tests (including the baseline MOT task) has been utilized previously and was proven effective in highlighting affected cognitive performance in the elderly population [70]. Our results are in strong alignment with those of Csipo and colleagues. MOT showed no difference between the two groups, confirming that participants of both groups had the same, sufficient sensorimotor skillset to perform the rest of the tasks. The DMS task revealed the same age-related increase in response latency, but not the decrease in correct responses in our study. The PAL, RVP, and SWM tasks indicated the same reduction of performance in the elderly group. On the other hand, participants in our aging cohort not only responded slower, but also with worse performance when compared to the young group. We found response latency increased in the elderly group during the PRM task too, unlike in Csipo et al. [70]. Overall, three conclusions can be drawn from the behavioral outcomes. First, in line with previous research, we confirmed that this particular set of cognitive tests is suitable and sensitive for quantitatively capturing age-related decline in multiple cognitive domains. Second, response time was affected in elderly individuals for practically all tasks where it was assessed (except for MOT). Task performance was maintained in the case of DMS and PRM, challenging visual pattern matching/recognition and short-term visual memory. This implies that even though neural circuits required for task solving are already affected, in these domains elderly individuals could compensate for the decrease in function with increased processing time. Similar phenomenon has been observed in aging, e.g., in error perception and response inhibition tasks [71, 72]. Third, performance in the elderly group was significantly reduced in four tasks (PAL, RTI, RVP, and SWM, with also increased response latency in RTI and RVP), challenging a wide array of cognitive domains (see Table 1) apart from pattern matching. These results are also in line with previous findings [73, 74]. Notably, the RTI task put forward a similar challenge as MOT, only in a more controlled setting and also including a preparatory phase, however, reduced performance in the elderly (in both accuracy and response time) could only be observed in the former. For one, this is in line with previous findings that error rate of older individuals increases when forced to respond quickly [72]. Second, it could indicate that — in line with previous research [75, 76] — neural processes related to preparation and cued action (that were not assessed in our current study) are also affected by aging. From a qualitative standpoint, the SWM task was the most complex among the administered tests and adjusting for the redundancy of the output variables (e.g., the number of attempts naturally increase with the number of errors made), this was the task where we found the most differences in performance, regardless of the difficulty level (4, 6, 8, and 12 boxes). This further supports the notion that increased task complexity further deteriorates performance in the elderly [77], plausibly reflecting a diminished cognitive reserve.

### Fractal connectivity is reduced in healthy aging

From the FrC analysis the most prominent finding is a pronounced decrease in cross-spectral slope (and also univariate fractal scaling exponent) generally over the entire cortex (see Fig. 1). One consistent outcome of several previous studies investigating age-related alterations of FC is disconnectivity (see, e.g., Ferreira and Busatto [16] for a review), that can also be associated with impaired cognition [78]. However, it must be stressed that the cross-spectral exponent does not reflect the “strength” of coupling between two processes, but instead how the strength of the coupling changes over various time scales; therefore, these results cannot be compared directly. On the other hand, when we compared integrated cross-spectral power values (representing a more “traditional” measure of FC), we found no differences between the two groups, unlike previous studies (e.g., Vecchio, Miraglia [79]). This might be understood due to different analysis strategies: we only considered broadband spectral power and connection-wise comparisons with Bonferroni adjustment, while a more detailed analysis in separated frequency ranges and utilizing network theoretical measures would be more sensitive in revealing age-related alterations. Nevertheless, in this study, we focused especially on FrC and even though evaluation of other FC approaches remain a task for future work, our results verify the importance of assessing FrC, as it can reveal age-related alterations that a conventional connectivity approach could not. Relatedly, to the best of our knowledge, no previous study investigated FrC in aging and how it is related to cognitive performance. Stylianou and colleagues recently proposed a method termed bivariate focus-based multifractal formalism (BFMF) for assessing multifractal connectivity in the time domain [58]. Even though BFMF — unlike MRCSA — assesses FrC in the time domain, it is established that the obtained measures are equivalent [80]. In their subsequent studies, Stylianou and coworkers employed BFMF to show that FrC changes during a visual pattern recognition paradigm [50] as well as it is affected in Parkinson’s Disease and responds to dopaminergic treatment [81]. Even though these results imply the association between FrC and cognitive performance (see, e.g., Fig. 4 in Stylianou, Kaposzta [81]), none of these studies compared FrC of young and healthy groups explicitly. On a different note, albeit we found marked age-related differences in FrC, the exact neurophysiological basis of this neural phenomenon — and fractal neural dynamics in general — is yet very poorly understood. According to the neural network oscillator model, slower fluctuations are generated by larger neuronal assemblies, and thus the $1/f$ nature of neural activity occurs as a superposition of incoming signaling from neuronal populations of varying sizes [82, 83]. Extending this concept, a reduced cross-spectral exponent might indeed reflect that disconnectivity develops in the aging brain affecting neuronal populations of varying sizes. Another popular theory links scale-free neural activity to a state of self-organized criticality [84] in the brain, allowing it to undergo global reorganization quickly in response to external stimuli [85, 86]. According to this notion, a change in spectral slope might reflect altering balance in incoming excitatory and inhibitory stimuli [87, 88]. Such a regional imbalance might indeed result in a desynchronization of brain regions spanning multiple frequency bands [89]; however, confirmation of this hypothesis requires further research. The right panel of Fig. 1 indicates the involvement of mostly the frontal and temporal regions, with most connections with altered spectral slopes also linked to those regions. This might be explained by the fact that the univariate spectral slope — or equivalently, the Hurst exponent — can indeed define the bivariate scaling exponent [90]. Accordingly, in line with previous studies, the obtained results indicate the involvement of the frontal and temporal lobes, or functionally, the frontotemporal network in aging.

### Associations between fractal connectivity and cognitive performance in elderly individuals

We found most associations between FrC and performance in the RVP and PAL tasks (see Figs. 2 and 3). RVP challenges sustained attention, and it is quite similar in its design to the widely popular n-back working memory paradigm [91]; only subjects must keep one (or more) fixed sequences in their working memory throughout a run, without updating it with every trial. It has been argued previously that working memory is one of the first cognitive domains to be affected by aging [92]; therefore, it is hardly surprising that we found most associations with a very similar task. Furthermore, regional spectral slopes of frontal and prefrontal regions also showed associations with RVPA, in line with previous research indicating the role of these cortical areas (such as the dorsolateral prefrontal cortex) in working memory and sustained attention [93, 94]. A similar pattern was found in relation to the number of attempts in the PAL task, challenging visual memory and learning. Age-related reorganization of cortical areas responsible for visual memory — overlapping with those found in our study — has been described previously, although without showing any difference in performance between young and elderly participants [95]. On the contrary, our results indicate that long-term coupling between frontal and parietal cortical areas is impediment to maintaining visual memory. Specifically, auto- and cross-spectral slopes were found reduced in aging, as discussed previously. On the other hand, we found an inverse relationship (see Fig. 2) between spectral slopes and RVP scores (the higher, the better) and a positive correlation (see Fig. 3) between those and the number of PAL attempts (the lower, the better). These results are slightly surprising, as young control individuals had overall better performance in most tasks, while also higher spectral exponents, and thus one might associate higher ${\beta }_{X}$ and ${\beta }_{X,Y}$ values with better cognitive capabilities. However, it must be stressed that we found spectral slopes and cognitive scores uncorrelated for almost all cases in the young group. Therefore, such a relationship cannot be generally established and thus the correlations found in the elderly group cannot be discredited. Instead, based on the data obtained we can speculate that reduction in ${\beta }_{X}$ and ${\beta }_{X,Y}$ might reflect a compensatory mechanism for ongoing (yet unknown) processes resulting in reduced cognitive capabilities. Nevertheless, resolution of these questions requires further, more elaborate research, beyond the limitations of the current study, as detailed below.

## Limitations and future perspectives

One of the biggest limitations of the current study comes from the limited spatio-temporal resolution of the utilized EEG system, when 14 cortical regions were monitored at a 256 Hz temporal sampling. Hence, one of our future goals is to repeat the current study using an EEG system with higher resolution in both the electrode space and time. A denser electrode montage would not only provide a more even (and more detailed) coverage of the entire cortex, but also allow for source reconstruction [96] in order to draw conclusions on the role of more precisely localized anatomical regions. On the other hand, the frequency range in our current analysis was limited to 2 to 22.5 Hz, due to the effects of the resampling scheme on filtered signals (see the Supplementary material in [40] for further ramifications). It has been shown that neural signals can indeed have multiple scaling ranges with different spectral exponents [43, 59, 97]; however, this can only be assessed when a broad range of time scales is available. Therefore, a higher temporal sampling would allow for exploring such phenomena as well, besides providing a better reconstruction of the power spectrum.

It must be stressed that power-law (fractal) scaling is not a universal property of dynamic processes, and in fact its presence has to be verified statistically [98]. Indeed, the fractal nature of EEG connectivity dynamics had been verified rigorously in past studies, even when it was reconstructed from sliding window analysis [53, 54] or directly assessed, similarly to MRCSA [58]. More importantly, Stylianou and colleagues investigated fractal connectivity in healthy elderly adults [81] and confirmed that most functional connections indeed expressed fractal scaling. Therefore, as statistical evaluation of power-law scaling is usually achieved by surrogate data testing (see, e.g., Racz et al. [53]) and MRCSA is a computationally expensive technique, in this study we did not test for the presence of fractality explicitly and instead we took its presence as granted based on the findings of previous studies conducted in both young [50] and elderly [81] populations.

Relatedly, we only identified resting-state FrC correlates of cognitive functions; however, we did not assess how FrC changes in response to cognitive stimulation, i.e., when solving a task. Our recording pipeline included EEG data collection while subjects performed 3 different cognitive tasks: the widely used n-back working memory task [32, 91], a visual pattern recognition task [29, 50], and a spatial navigation task in a virtual environment. Analysis and discussion of these data are beyond the scope of the current study; however, our immediate future goal is to evaluate these recordings for a more complete understanding of the relationship between FrC, cognitive performance, and aging. Furthermore, even though MRCSA provides unbiased estimates of spectral scaling exponents, it is computationally expensive and thus unsuitable for certain applications, such as online cognitive state monitoring. Other alternatives might be considered in FrC analysis for such purposes, such as the recently introduced real-time detrended cross-correlation analysis [99]. Finally, in this initial study, we focused explicitly on healthy aging, in order to identify biomarkers of this state when compared to young control individuals. However, another important research endeavor is to discriminate changes occurring naturally in aging from those characteristic of pathological conditions such as mild cognitive impairment or Alzheimer’s Disease. Therefore, our future aims also include investigating FrC and its associations to cognition not only in healthy aging but also in relevant clinical conditions, such as those mentioned above.

## Conclusions

In this study, we investigated resting-state fractal brain connectivity in healthy elderly individuals and young control adults and explored its associations with performance in various cognitive domains. Cognitive assessment robustly indicated increased response latency and impaired performance in the elderly group over multiple tasks that were accompanied by a global decrease in regional and cross-regional spectral exponents. Correlation analyses revealed that fractal connectivity dynamics were inversely related to performance in elderly individuals, with the reduction in FrC likely reflecting an attempt at compensating for impaired cognitive capabilities. Our results are the first to identify age-related correlates of fractal connectivity and their relation to cognitive functions, and thus will pave the way for future research aiming at exploiting these markers for screening, monitoring, or even intervention purposes.

### Supplementary Information

Below is the link to the electronic supplementary material.

Supplementary file1 (DOCX 772 KB)

## Data Availability

In this study, we collected electroencephalography recordings and neuropsychological test scores. Deidentified data that support the findings of this study will be made available on PhysioNet.org (project entitled “Cognitive and neurophysiological measurements in healthy young and elderly participants”) and can also be requested from the corresponding author.
