# Supplementary material for: Resting-state fractal brain connectivity is associated with impaired cognitive performance in healthy aging
Source: GeroScience. 2023 Jul 17;46(1):473–89. doi: 10.1007/s11357-023-00836-z (PMC10828136; doi:10.1007/s11357-023-00836-z)
Supplement: Supplementary file 1 — Supplementary file1 (DOCX 772 KB) [file 11357_2023_836_MOESM1_ESM.docx]

Supplementary material – Resting-state fractal brain connectivity is associated with impaired cognitive performance in healthy aging

**Akos Czoch^1^, Zalan Kaposzta^1^, Peter Mukli^1,2,3,4^, Orestis Stylianou^1,5,6,7^, Andras Eke^1,8^ and Frigyes Samuel Racz^1,9,10,*^**

*^1^Department of Physiology, Semmelweis University, Budapest, Hungary*

*^2^Oklahoma Center for Geroscience and Healthy Brain Aging, University of Oklahoma Health Sciences Center, Oklahoma City, OK, United States of America*

*^3^Vascular Cognitive Impairment and Neurodegeneration Program, Department of Neurosurgery, University of Oklahoma Health Sciences Center, Oklahoma City, OK, United States of America*

*^4^International Training Program in Geroscience, Doctoral School of Basic and Translational Medicine/Department of Public Health, Semmelweis University, Budapest, Hungary*

*^5^Institute of Translational Medicine, Semmelweis University, Budapest, Hungary*

*^6^Berlin Institute of Health at Charité, University Hospital Berlin, Charitéplatz 1, 10117 Berlin, Germany*

*^7^Department of Neurology with Experimental Neurology, Charité-University Hospital Berlin, Corporate Member of Freie Universität Berlin and Humboldt Universität zu Berlin, Berlin, Germany*

*^8^Department of Radiology and Biomedical Imaging, Yale University School of Medicine, New Haven, CT, United States of America*

*^9^Department of Neurology, Dell Medical School, The University of Texas at Austin, Austin, TX, United States of America*

*^10^Mulva Clinic for the Neurosciences, Dell Medical School, The University of Texas at Austin, Austin, TX, United States of America*

****Correspondence****: Frigyes Samuel Racz, MD, PhD; Department of Physiology, Semmelweis University; 37-47 Tuzolto Street, 1094 Budapest, Hungary; email:* [*racz.frigyes@med.semmelweis-univ.hu,*](mailto:racz.frigyes@med.semmelweis-univ.hu) [*fsr324@austin.utexas.edu*](mailto:fsr324@austin.utexas.edu)

# Results

## Behavioral results

**Supplementary Table S1** presents all CANTAB output measures that were found to be different between young and elderly groups. $E[young]$ and $E[elderly]$ denotes the expected value (mean or median) in the young and elderly groups, respectively, depending on the normality of the data. Brief descriptions are provided for all output measures, while detailed explanations can be found at <https://www.cambridgecognition.com/cantab/>. Numerical elements in the descriptions refer to various versions of the given task, e.g., DMSML4 indicates mean correct latency in the delayed matching to sample task after 4 seconds, in contrast to DMSML12 indicating mean correct latency after 12 seconds, or PALTA4 indicating number of total attempts in the paired associates learning task for 4 patterns, in contrast to PALTA6 referring to the number of total attempts with 6 patterns. All *p*-values reported in **Supplementary Table S1** are adjusted using False Discovery Rate correction of Benjamini and Hochberg [1].

**Supplementary Table S1.** Significant differences in CANTAB scores between young and elderly groups. DMS: Delayed Matching to Sample; PAL: Paired Associates Learning; PRM: Pattern Recognition Memory; RTI: Reaction Time; RVP: Rapid Visual Information Processing; SWM: Spatial Working Memory; SD: standard deviation; CL: correct latency.

| Task | Measure | Definition | $E\left[ young \right]$ | $E\left[ elderly \right]$ | *p*-value |
| --- | --- | --- | --- | --- | --- |
| DMS | DMSL4SD | Correct latency SD | 827.6 | 1456.7 | 0.0427 |
|  | DMSMDL | Median correct latency | 2284 | 3459 | 0.0122 |
|  | DMSMDL12 | Median correct latency 12 | 2513.3 | 3869 | 0.0097 |
|  | DMSMDLAD | Median correct latency all | 2287.8 | 3586 | 0.0241 |
|  | DMSMDLS | Simultaneous DMSMDL | 2252.5 | 3238.9 | 0.0010 |
|  | DMSML | Mean correct latency | 2820.4 | 3677.5 | 0.0270 |
|  | DMSML12 | Mean correct latency 12 | 2846.1 | 4322 | 0.0189 |
|  | DMSML4 | Mean correct latency 4 | 2475.7 | 3634.2 | 0.0218 |
|  | DMSMLAD | Mean correct latency all | 2487 | 3956.7 | 0.0184 |
|  | DMSMLS | Simultaneous DMSML | 2403.7 | 3276.6 | 0.0033 |
| PAL | PALFAMS28 | First attempt memory score | 16.4517 | 11.9474 | 0.0004 |
|  | PALFAMS28% | PALFAMS28 percentile | 66.5 | 44.0526 | 0.0197 |
|  | PALFAMS28Z | PALFAMS28 Z-score | 0.6242 | -0.1684 | 0.0178 |
|  | PALMETS28 | Mean errors to success | 1 | 2 | 0.0013 |
|  | PALTA28 | Total attempts all | 6 | 8 | 0.0032 |
|  | PALTA4 | Total attempts 4 | 1 | 1 | 0.0241 |
|  | PALTA6 | Total attempts 6 | 1 | 2 | 0.0013 |
|  | PALTE28 | Total errors all | 5 | 13.1579 | 0.0014 |
|  | PALTE4 | Total errors 4 | 0 | 0 | 0.0251 |
|  | PALTE6 | Total errors 6 | 0 | 3 | 0.0012 |
|  | PALTE8 | Total errors 8 | 4.0417 | 8.1579 | 0.0314 |
|  | PALTEA12 | Adjusted PALTE 12 | 5.5 | 12 | 0.0185 |
|  | PALTEA28 | Adjusted PALTE all | 5 | 13.1579 | 0.0013 |
|  | PALTEA4 | Adjusted PALTE 4 | 0 | 0 | 0.0245 |
|  | PALTEA6 | Adjusted PALTE 6 | 0 | 3 | 0.0010 |
|  | PALTEA8 | Adjusted PALTE 8 | 4.0417 | 8.1579 | 0.0308 |
| PRM | PRMMCLD | Mean correct latency delayed | 1667.1 | 2082.1 | 0.0123 |
|  | PRMMCLI | Mean CL immediate | 1516.4 | 1900.7 | 0.0142 |
|  | PRMMDCLD | Median CL delayed | 1531 | 1927.8 | 0.0033 |
|  | PRMMDCLI | Median CL immediate | 1280.3 | 1744 | 0.0015 |
| RTI | RTIFESI | Error score 5 (inaccurate) | 0 | 0 | 0.0244 |
|  | RTIFMDRT | Median reaction time 5 | 334.3542 | 383.7895 | 0.0022 |
|  | RTIFMRT | Mean reaction time 5 | 341.5054 | 391.4468 | 0.0022 |
|  | RTIFRTSD | Reaction time 5 SD | 36.5422 | 46.9 | 0.0228 |
| RVP | RVPA | RVP A prime measure | 0.9398 | 0.89871 | 0.0143 |
|  | RVPA% | RVPA percentile | 59.9583 | 36.2632 | 0.0126 |
|  | RVPAZ | RVPA Z-score | 0.3454 | -0.4242 | 0.0186 |
|  | RVPMDL | Median response latency | 411.5 | 528 | 0.0002 |
|  | RVPML | Mean response latency | 466.6207 | 568 | 0.0010 |
|  | RVPPH | Probability of hit | 0.7431 | 0.6140 | 0.0423 |
|  | RVPTH | Total hits | 40.1250 | 33.1579 | 0.0415 |
|  | RVPTM | Total misses | 13.8750 | 20.8421 | 0.0407 |
| SWM | SWMBE12 | Between errors 12 | 14 | 35 | 0.0082 |
|  | SWMBE4 | Between errors 4 | 0 | 2 | 0.0192 |
|  | SWMBE468 | Between errors 4-6-8 | 5 | 19 | 0.0181 |
|  | SWMBE6 | Between errors 6 | 0 | 6 | 0.0277 |
|  | SWMS | Strategy score 6-8 | 6.7083 | 8.9474 | 0.0302 |
|  | SWMS6 | Strategy score 6 | 3 | 4 | 0.0139 |
|  | SWMSX | Strategy score 6-12 | 11.375 | 15.5263 | 0.0241 |
|  | SWMTE12 | Total errors 12 | 14 | 35 | 0.0113 |
|  | SWMTE4 | Total errors 4 | 0 | 2 | 0.0186 |
|  | SWMTE468 | Total errors 4-6-8 | 6.375 | 15.7895 | 0.0086 |
|  | SWMTE6 | Total errors 6 | 0 | 6 | 0.0312 |
|  | SWMWE8 | Within errors 8 | 0 | 0 | 0.0284 |

## Correlations between cognitive function and fractal connectivity

**Supplementary Figure S1** shows correlations found between the probability of hit during the rapid visual processing task (RVPPH) and auto- and cross-spectral slopes in the elderly group. Since the probability of hit is strongly associated with the overall performance in the task, these results are almost identical with those shown on **Figure 2** of the main manuscript. Notably, **Figure 2** might raise the concern that correlations are driven/biased by an outlier participant, however this notion is disproven by the scatter plots on the left panel of **Supplementary Figure S1**, showing and even distribution of data.


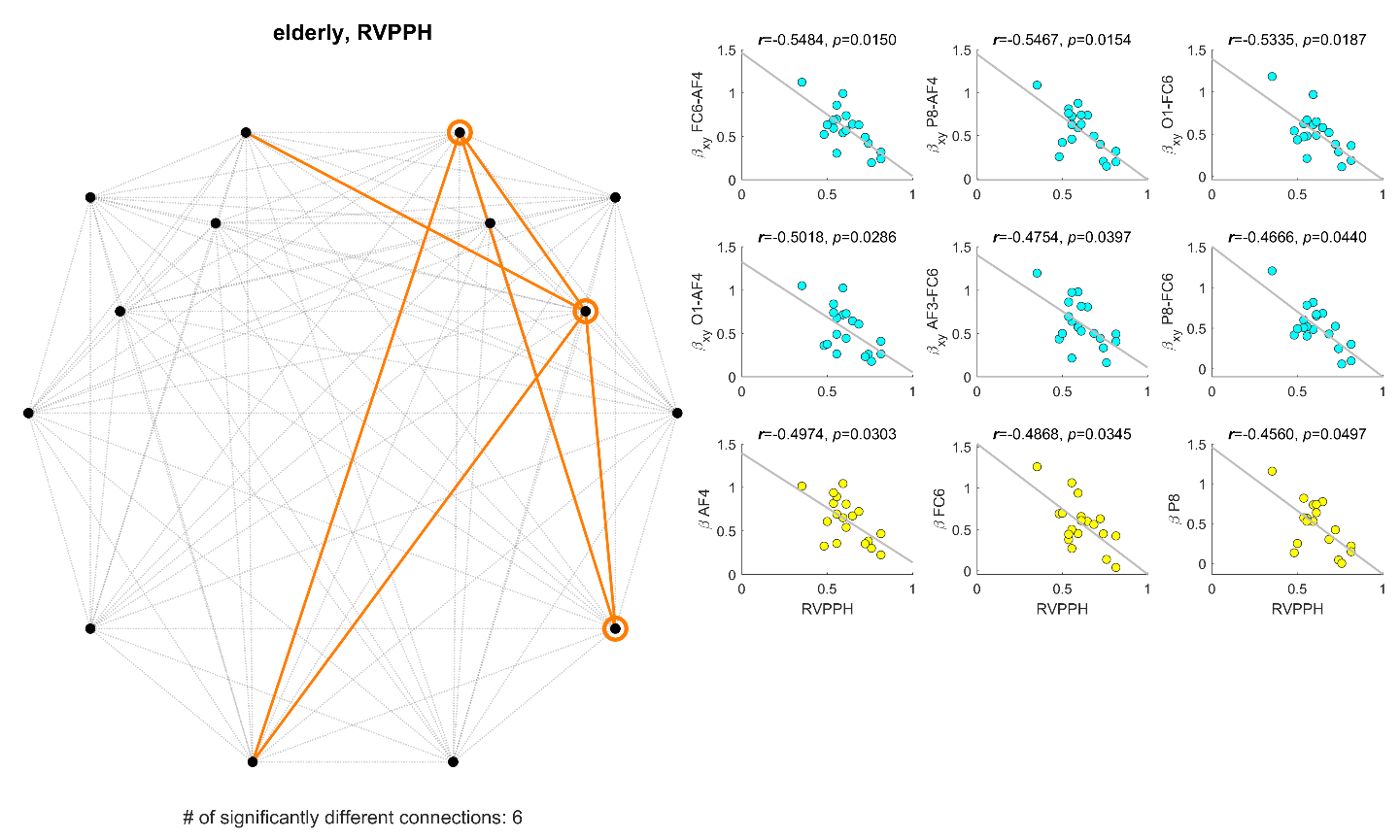


**Supplementary Figure S1.** Significant correlations between spectral exponents and Probability of Hit in Rapid Visual Processing (RVPPH). Left panel indicates the locations (circles) and connections (orange lines) where $\beta_{X}$ or $\beta_{X,Y}$ expressed significant correlation with RVPPH scores. Subplots of the right panel show the individual scatterplots for all relationships (yellow: $\beta_{X}$ vs. RVPPH, blue: $\beta_{X,Y}$ vs. RVPPH).

***Supplementary Figure S2*** shows all connections in both groups whose cross-spectral slopes were found correlated with any CANTAB score different between young and old populations. Notably, only two CANTAB measures (DMSL4SD and PALMETS28) expressed a significant relationship with fractal connectivity (four connections in total) in the young group. Fractal connectivity in the elderly population was found associated with a broader range of cognitive measures, however most of these were sporadic, with the exception of indices of rapid visual processing (RVP) task. It should be noted that *p*-values were not adjusted throughout the correlation analyses, therefore most of the sporadic correlations are most likely reflect statistical chance instead of a true relationship between neural activity and cognitive performance.


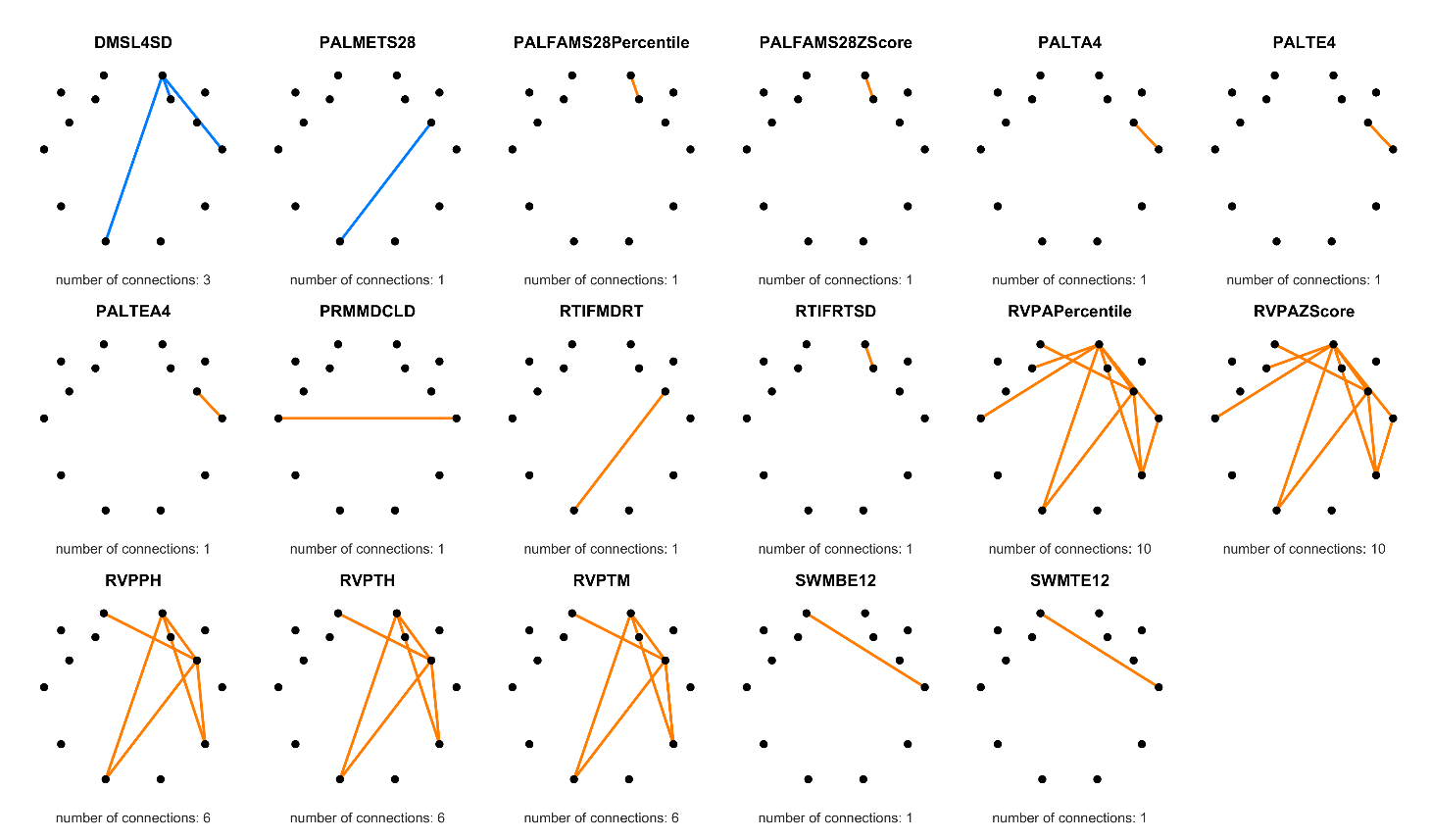


**Supplementary Figure S2.** Significant correlations between $\beta_{X,Y}$ of connections and CANTAB scores. Each subplot indicates the connections whose $\beta_{X,Y}$ was found significantly correlated with the CANTAB output measure indicated above the panel. Blue and orange colors indicate correlations found in the young and elderly groups, respectively. For output measure definitions, please refer to **Supplementary Table S1**.

Similarly to ***Supplementary Figure S2***, ***Supplementary Figure S3*** presents all regions in both cohorts whose monofractal exponents were found significantly correlated with CANTAB scores. Notably, in the young group significant associations were only found in case of 3 measures (DMSL4SD, DMSML4 and RTIFRTSD). Not unlike cross-spectral exponents, correlations between $\beta_{X}$ and cognitive performance in the elderly group were found sporadic for most tasks except RVP.


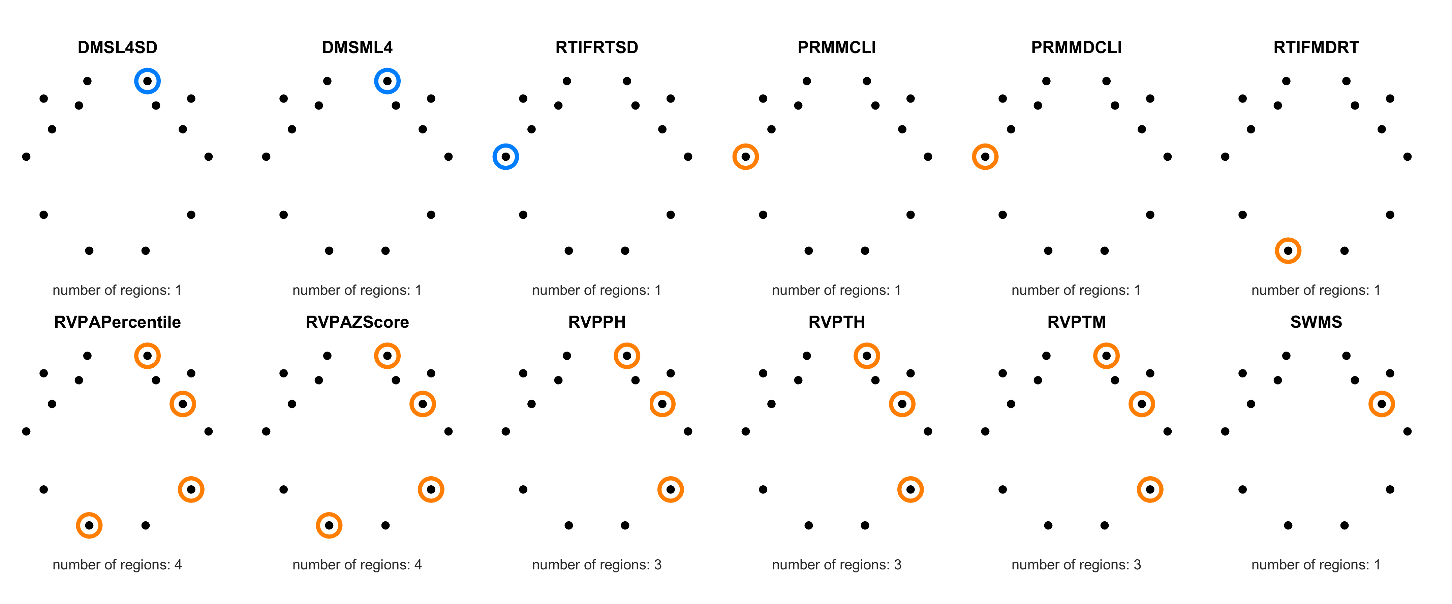


**Supplementary Figure S3.** Significant correlations between $\beta_{X}$ of cortical regions and CANTAB scores. Each subplot indicates the EEG electrode location whose $\beta_{X}$ was found significantly correlated with the CANTAB output measure indicated above the panel. Blue and orange circles indicate correlations found in the young and elderly groups, respectively. For output measure definitions, please refer to **Supplementary Table S1**.

# References

1. Benjamini, Y. and Y. Hochberg, *Controlling the False Discovery Rate - a Practical and Powerful Approach to Multiple Testing.* Journal of the Royal Statistical Society Series B-Statistical Methodology, 1995. **57**(1): p. 289-300.
